# *Xylella* phage MATE 2: a novel bacteriophage with potent lytic activity against *Xylella fastidiosa* subsp. *pauca*

**DOI:** 10.3389/fmicb.2024.1412650

**Published:** 2024-05-28

**Authors:** Miloud Sabri, Kaoutar El Handi, Orges Cara, Angelo De Stradis, Franco Valentini, Toufic Elbeaino

**Affiliations:** ^1^International Centre for Advanced Mediterranean Agronomic Studies (CIHEAM of Bari), Valenzano, Italy; ^2^Department of Soil, Plant and Food Science, University of Bari, Bari, Italy; ^3^Institute for Sustainable Plant Protection (IPSP), National Research Council of Italy (CNR), University of Bari, Bari, Italy; ^4^Institute for Sustainable Plant Protection (IPSP), National Research Council of Italy (CNR), Portici, Italy

**Keywords:** *Xylella fastidiosa*, bacteriophage, high-throughput sequencing, genome characterization, biocontrol

## Abstract

*Xylella fastidiosa* (*Xf*) is a major phytosanitary threat to global agricultural production. The complexity and difficulty of controlling *Xf* underscore the pressing need for novel antibacterial agents, i.e., bacteriophages, which are natural predators of bacteria. In this study, a novel lytic bacteriophage of *Xf* subsp. *pauca*, namely *Xylella* phage MATE 2 (MATE 2), was isolated from sewage water in southern Italy. Biological characterization showed that MATE 2 possessed a broad-spectrum of antibacterial activity against various phytobacteria within the family *Xanthomonadaceae*, a rapid adsorption time (10 min), and high resistance to a broad range of pH (4–10) and temperatures (4–60°C). Most importantly, MATE 2 was able to suppress the growth of *Xf* subsp. *pauca* cells in liquid culture for 7 days, demonstrating its potential as an effective antibacterial agent against *Xf*. The genomic and electron microscopy analyses revealed that MATE 2 is a new species tentatively belonging to the genus *Carpasinavirus* within the class *Caudoviricetes*, with an isometric capsid head of 60 ± 5 nm along with a contractile tail of 120 ± 7.5 nm. Furthermore, the high-throughput sequencing and *de novo* assembly generated a single contig of 63,695 nucleotides in length; representing a complete genome composed of 95 Open Reading Frames. Bioinformatics analysis performed on MATE 2 genome revealed the absence of lysogenic mediated genes, and genes encoding virulence factors, antibiotic resistance, and toxins. This study adds a new phage to the very short list of *Xf*-infecting lytic phages, whose *in-vitro* antibacterial activity has been ascertained, while its efficacy on *Xf*-infected olive trees in the field has yet to be determined.

## 1 Introduction

Bacteriophages, often known simply as phages, are viruses that specifically infect and kill bacteria; hence, the term “bacteriophages” means “bacteria eaters” (Chanishvili, 2016). Phages are ubiquitous, existing in various environments, including deserts, polar regions, freshwater, high-salt environments, oceans, soils, or other organisms, and their number is estimated at approximately 10^34^, making them the most abundant and diverse biological entities in the biosphere (Peng et al., 2023). Discovered more than a century ago, research on phages has recently advanced considerably, improving our understanding on their biology, genetics, ecological function, and impact on biodiversity. Indeed, the current antimicrobial resistance (AMR) crisis has reignited interest in phage therapy as a promising alternative for controlling bacteria in humans, animals, foods, and plants (Liu et al., 2024). Compared with conventional antimicrobials, phages display a numerous of advantageous characteristics, including self-replicating nature, high host-bacteria specificity, low environmental impact, ease of discovery, harmlessness to eukaryotes, high efficiency at low multiplicity of infection (MOI), low cost and simple process for preparation, and effectiveness against multidrug-resistant bacteria (MDR) and in inhibiting the formation of biofilm (Loc-Carrillo and Abedon, 2011; Peng et al., 2023). Moreover, in agricultural applications, the efficient translocation of phages within plants through the vascular system, coupled with their high environmental stability, render them as viable choice for controlling plant pathogenic bacteria, particularly those colonizing xylem vessels, i.e., *Xf* (Buttimer et al., 2017).

*Xylella fastidiosa* is one of the most dangerous plant pathogens worldwide, posing a significant threat to various economically crucial crops. In Europe, the presence of *Xf* was first identified in Apulia (Italy) in 2013 on olives, resulting in catastrophic economic damage, mainly in olive groves (Castro et al., 2021). Its rapid spread to around 54,000 hectares and the death of 10 million olive trees in southern Italy have raised alarming concerns across the Mediterranean basin (Bajocco et al., 2023). Despite the substantial investment and research efforts undertaken to combat *Xf*, effective and sustainable control of this pathogen remains a challenge. Therefore, there is an urgent need to explore novel antibacterial agents for controlling *Xf*.

Within the different possibilities available in the field of biological control, virulent (lytic) phages are undoubtedly promising candidates for contrasting *Xf* infections. Currently, significant efforts are underway in the development and commercialization of various phage products targeting a range of plant pathogenic bacteria, including various strains of *Xanthomonas* and *Ralstonia solanacearum* (Erdrich et al., 2022). In this context, OmniLytics^™^ has successfully introduced several phage products branded as AgriPhage^™^ to control *Pseudomonas syringae* and *Xanthomonas campestris* on tomato and pepper plants, along with other plant diseases (Reuter and Kruger, 2020). Furthermore, a commercial phage-based bactericide (XylPhi-PD) against Pierce’s Disease (PD) of grapevines, caused by *Xf* subsp. f*astidiosa,* has been developed by A&P Inhpatec.^1^ Additionally, a research team from Texas A&M University reported for the first time the development of a cocktail composed of four lytic phages with great potential for biocontrol of PD (Das et al., 2015). Building upon this research trajectory, and due to the lack of lytic phages targeting *Xf* subsp., *pauca*, our focus has been on isolating *Xf* phages, specifically targeting the subsp. *pauca*, laying the foundation for potential biocontrol products.

## 2 Materials and methods

### 2.1 Bacterial isolates and growth conditions

*Xylella fastidiosa* subsp. *pauca* strain A0PT1, isolated from olive trees affected by the olive quick decline syndrome (OQDS) in southern Italy (Apulia region), was used in all our experiments. The strain was stored in phosphate buffered saline (PBS) supplemented with glycerol (50%) and maintained at −80°C. When needed, aliquots were cultured in buffered charcoal yeast extract (BCYE) agar plates (Wells et al., 1981) and grown at 28°C for 10 to15 days. Cell suspensions were prepared in PBS and adjusted to 10^8^ CFU/mL (OD_600_ ≅ 0.32). The *Xanthomonas* strains used for bacteriophage isolation, as well as the bacteria listed in the Table 1 used for phage host range determination, were grown either at 28°C in liquid yeast extract peptone glucose broth (YPG) (5.0 g/L yeast extract, 5.0 g/L peptone and 10.0 g/L glucose) or on yeast extract peptone glucose agar (YPGA, i.e., YPG supplemented with 1.5% agar).

**Table 1:** Bacterial strains used for phage host range determination in this study.

Species	Isolate	Host plant	Origin
*Xylella fastidiosa* subsp. *pauca*	IAMB A0PT1*	*Olea europaea*	Italy
*Xylella fastidiosa* subsp. *pauca*	IAMB OR1*	*Oleander nerium*	Italy
*Xylella fastidiosa* subsp. *pauca*	IAMB B3*	*Polygala myrtifolia*	Italy
*Xanthomonas campestris* pv. *campestris*	CFBP 1710	*Brassica oleracea* var. *botrytis*	France
*Xanthomonas albilineans*	CFBP 1943	*–*	Burkina Faso
*Xanthomonas albilineans*	CFBP 2523	*–*	Fiji
*Xanthomonas albilineans*	CFBP 1211	*–*	–
*Erwinia amylovora*	PGL Z1*	*Pyrus communis*	Italy
*Pseudomonas syringae* pv*. syringae*	CFBP 311	*Pyrus communis*	Indre et Loire-France
*Dickeya chrysanthemi* biovar *chrysanthemi*	CFBP 1346	*Chrysanthemum maximum*	Italy
*Pseudomonas savastanoi* pv *savastanoi*	CFBP 5050	*Olea europaea*	Portugal
*Agrobacterium vitis*	CFBP 2738	*Vitis vinifera*	Greece
*Agrobacterium larrymoorei*	CFBP 5473	*Ficus benjamina*	USA
*Agrobacterium rubi*	CFBP 5521	*Rubus sp.*	Germany
*Agrobacterium* sp. biovar 1	CFBP 5770	*Prunus persica*	Australia
*Agrobacterium* sp.	CFBP 2514	*Vitis vinifera*	Spain
*Paenibacillus rigui*	S55*	*Olea europaea*	Italy
*Bacillus subtilis*	L39*	*Olea europaea*	Italy
*Bacillus pumilus*	L36*	*Olea europaea*	Italy
*Pantoea agglomerans*	BA69*	*Nerium oleander*	Italy

CFBP: French Collection of Phytopathogenic Bacteria, Angers, France.

*Collection of CIHEAM-IAM, Bari, Italy.

### 2.2 Bacteriophage isolation and purification

The phage described in this study was isolated from a sewage water sample collected in April 2023, from the untreated influx point at the wastewater processing station of Bari (south of Italy). Briefly, 1 L of sewage water was passed through a filter paper of Grade 1, Dia. 75 × 100 mm (Whatman, Maidstone, UK) to remove large particles and thoroughly filtered with 0.22 μm filter (Merck, Rome, Italy) to remove cellular debris. The filtrate was centrifuged at 108,763 g (Rotor J50.2 Ti, Beckmann) for 1 h at 4°C to pellet phage particles. Pellets were resuspended in 2 mL phage buffer (100 mM Tris–HCl (pH 7.6); 10 mM MgCl_2_; 100 mM NaCl; and 10 mM MgSO_4_) and stored at 4°C.

Given the challenging slow-growing nature of *Xf* subsp. *pauca*, an optimized protocol for fast isolation of bacteriophages infecting this bacterium was developed. To directly select active bacteriophages against *Xf* subsp. *pauca*, 100 μL of pre-treated sample were added to 900 μL of *Xf* cells suspended in phage buffer at OD_600_ of 0.2, and the mixture was incubated at 28°C for phage adsorption during 10 min. The mixture was subsequently centrifuged at 10,000 *g* for 5 min to precipitate the phage-adsorbed bacteria, and the pellet was resuspended in 100 μL phage buffer. In addition, untreated *Xf* subsp. *pauca* suspension and *Xf* subsp. *pauca* treated with *Agrobacterium* phage PAT1 from our collection, which is inactive against *Xf* subsp. *pauca* (data not shown), were utilized as controls to ensure the absence of non-specific phage adsorption to *Xf* subsp. *pauca*. For phage enrichment, adsorbed phages were enriched on *Xf* subsp. *pauca* and on fast-growing bacteria phylogenetically close to *Xf,* i.e., *Xanthomonas albilineans* (*Xa*) and *Xanthomonas campestris* pv. *campestris* (*Xcc*), to facilitate and maximize our chances of isolating *Xf* subsp. *pauca*-infecting phages. The culture enrichments underwent incubation at 28°C, lasting 24 h for *Xanthomonas* strains and for 10 days for *Xf* subsp. *pauca*. Then, cultures with phages were centrifuged at 10,000 *g* for 5 min, and the supernatants were filtered (0.22-μm filter) and stored at 4°C. Phage was isolated and purified from filtrates using *Xcc* bacteria via the standard double agar overlay method (Kropinski et al., 2009). Single clear plaque-forming unit was transferred into 1 mL of phage buffer and this process was repeated three times to ensure the isolation of a single phage. To obtain high phage titer, 1 mL of *Xcc* culture (OD_600_ = 0.2) was inoculated in 500 mL YPG broth medium and 1 mL of purified phage was added, and the mix was then incubated for up to 24 h at 28°C. Amplified phages were filtered through 0.22-μm filters, concentrated by high-speed centrifugation at 108,763 g for 2.5 h, resuspended in 2 mL of phage buffer, and stored at 4°C for further analysis. The phage titer was determined through a double-layer assay.

### 2.3 Transmission electron microscopy

To scrutinize the morphological and lytic properties of the purified phage (assigned MATE 2 here for brevity), a culture of *Xf* subsp. *pauca* was subjected to challenge with MATE 2 (MOI = 1) at room temperature for 1 h. Representative images of the phage and bacterial cells were taken at 10, 30, and 60 min post-infection (pi) via TEM (FEI MORGAGNI 282D, United States) using the dip method. Briefly, carbon-coated copper/rhodium grids underwent a 2-min incubation period with either the phage alone or the phage-treated cells, followed by rinsing with 200 μL of distilled water. Negative staining was obtained by immersing the grids in 200 μL of a 0.5% w/v UA-Zero EM stain solution (Agar-Scientific Ltd., Stansted, UK), and observed under an accelerating voltage of 80 kV.

### 2.4 DNA extraction, complete genome sequencing, and bioinformatics analysis

Genomic DNA of MATE 2 was extracted from a high-titer stock of phage particles at ~10^10^ PFU/mL using a DNeasy Plant Extraction kit following the manufacturer’s protocol (Qiagen, Milan, Italy). The extracted DNA was quantified using the NanoDrop^™^ One/OneC Microvolume UV–Vis Spectrophotometer (ThermoFisher Scientific, Waltham, MA, United States). Subsequently, 500 ng of purified genomic DNA was sent for Illumina sequencing (2 × 150 bp paired-end mode) (Eurofins Genomics, Germany). The reads were quality checked and trimmed and *de novo* assembled using the Tadpole tool with different k-mers (Geneious Prime 2024.0.3, San Diego, CA, United States). The ORFs functions were annotated with Geneious, and the predictions of antibiotic resistance genes, acquired virulence genes, and toxin-encoding genes were assessed by CGE.^2^ The complete genome sequence of MATE 2 was deposited at GenBank and a circular map of the genome and phylogenetic tree based on proteomic analysis were constructed by utilizing VipTree (Nishimura et al., 2017).

### 2.5 Spot assay

To evaluate the bacteriolytic effect of MATE 2 on *Xf* subsp. *pauca*, spot assay was carried out as follows: two drops of *Xf* subsp. *pauca* cells in suspension (10^8^ CFU/mL), each containing 30 μL, were positioned at the upper portion of the BCYE agar plates, with approximately 1.5 cm between them. These drops were allowed to let down to the opposite side, forming two parallel rows of *Xf* cultures. Subsequently, 10 μL of MATE 2 (10^8^ PFU/mL) were administered to the upper part of the rows of *Xf* cultures after being dried under the laminar flow hood. Phage buffer was used as a negative control. This experiment was conducted with three independent replicates.

### 2.6 Bacterial reduction assay

To investigate the phage’s ability to inhibit the growth of *Xf* subsp. *pauca* in liquid growth medium, a suspension *Xf* subsp. *pauca* (200 μL) was challenged with 200 μL of phage MATE 2 (MOI = 1). The mixture was then inoculated in 2 mL of BCYE broth and incubated at 28°C for 7 days. During incubation, optical density measures (each day) at OD_600_ were taken using the NanoDrop^™^ One/OneC Microvolume UV–Vis Spectrophotometer.

### 2.7 Fluorescence microscopy

Fluorescence microscopy provides accurate assessment of the bacterial lysis process by enumerating intact and permeable cell populations at different time intervals. In this context, an aliquot of *Xf* subsp. *pauca* culture was incubated with MATE 2 (MOI = 1) at room temperature. The LIVE/DEAD^®^ BacLight^™^ viability kit (ThermoFisher Scientific, Milan, Italy) was employed according to the manufacturer’s recommendations to assess the viability of MATE 2-treated *Xf* cells at 0, 3, 6, and 24 h post-infection. Photomicrographs were captured using a Nikon E800 microscope equipped with fluorescein isothiocyanate (480/30 excitation filter, DM505 dichroic mirror, 535/40 emission filter) and tetramethyl rhodamine isothiocyanate (546/10 excitation filter, DM575 dichroic mirror, 590 emission filter) fluorescence filter sets.

### 2.8 Host range analysis

Host range of MATE 2 was determined using the phage sensitivity spot test as follows: bacterial strains listed in Table 1 (except for *Xf* subsp. *pauca* strains, which were cultured in BCYE plates as described in spot assay) were cultured at 28°C on YPGA plates for up to 2 days. Then, the cultures were suspended in sterile distilled water and 300 μL of bacterial suspension (OD_600_ = 0.2) were evenly spread on YPGA plates and allowed to dry. Subsequently, drops of 10 μL of MATE 2 solution (10^8^ PFU/mL) were spotted onto the surface of the plates. Spots were dried at room temperature and the plates cultured for up to 2 days at 28°C. The presence of a clear zone was recorded as the strain being susceptible to MATE 2.

### 2.9 Temperature and pH stability

The thermal stability of MATE 2 was assessed by incubating 100 μL of phage suspension (~10^8^ PFU/mL) at 4, 28, 40, 50, and 60°C for 1 h. Following the incubation period, a serial dilution was made with phage buffer, and phage titers were determined using *Xcc* bacteria via the double agar overlay method. To assess pH stability, 100 μL of the phage suspensions were added to 900 μL of sterile-filtered YPG medium that was pH adjusted using 1 M NaOH or 1 M HCl and incubated at 28°C for 1 h. Subsequently, a serial dilution was made with phage buffer, and phage titers were determined using double agar overlay method.

## 3 Results

### 3.1 Morphology and host range

**Figure 1:**
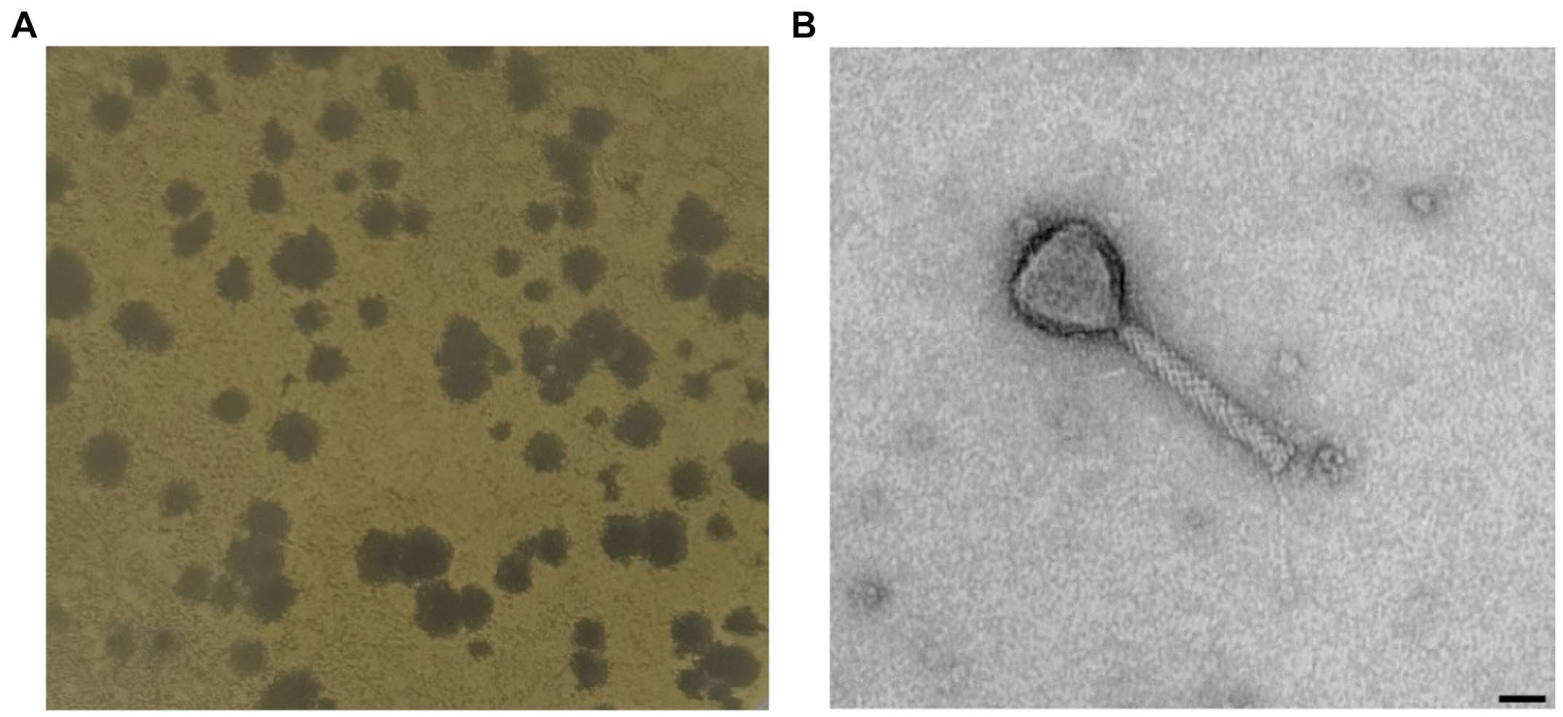
**(A)** Plaque morphology of MATE 2 formed in double-layer agar plate with *Xanthomonas campestris* as host strain. **(B)** Transmission electron microscopy image of MATE 2 showing a particle with an isometric capsid head and a long contractile tail, scale bar = 25 nm.

One novel discovered phage, hereafter referred to as *Xylella* phage MATE 2 (MATE 2), that infects *Xf* subsp. *pauca* has been isolated from sewage water in Bari, Italy. In double agar overlay assay, MATE 2 produced large (4–5 mm), round, and clear plaques on *Xcc* culture plates (Figure 1A). The morphological analyses using TEM imaging, revealed that MATE 2 resembles myrovirus phages (A1 morphotype) of the class *Caudoviricetes* that group all tailed bacterial and archaeal viruses with icosahedral capsids and a double-stranded DNA genome (Turner et al., 2023). The phage particle showed to have an isometric capsid head of 60 ± 5 nm along with a contractile tail of 120 ± 7.5 nm (Figure 1B).

For use of phage in biocontrol, host specificity is a key element to consider. To determine the host range of MATE 2, a range of different bacteria were tested for susceptibility, including beneficial bacteria, namely *Paenibacillus rigui*, *Bacillus subtilis*, *Bacillus pumilus*, and *Pantoea agglomerans* (Table 1). The results showed that MATE 2 had no lytic activity against the beneficial bacteria tested, suggesting its potential safe use without affecting beneficial bacteria possibly present in host plants. Furthermore, among the plant pathogenic bacteria tested, MATE 2 was able to produce a clear lysis zone against *Xcc* and all tested isolates of *Xf* subsp. *pauca* and *Xa*.

### 3.2 Genomic and phylogenetic analyses of MATE 2

**Figure 2:**
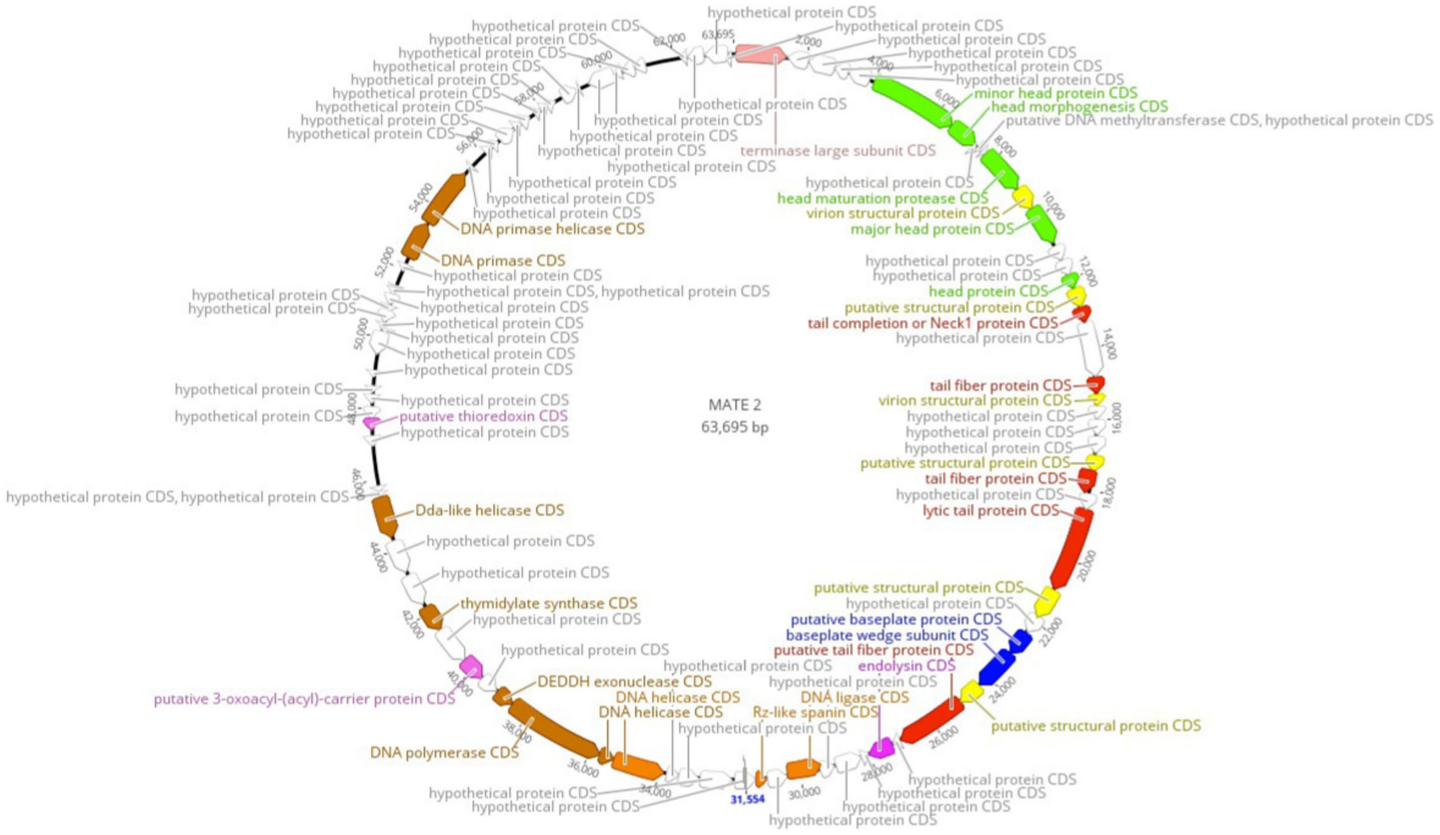
Circular genomic map of *Xylella* phage MATE 2 representing 95 ORFs encoded by the genome. Hypothetical proteins are displayed in gray and the predicted proteins with a signed functions are highlighted on the genomic map. The map was generated using Geneious Prime 2024.0.3.

Complete genome sequencing and *de novo* assembly of MATE 2 revealed a double-stranded DNA genome of 63,695 base pairs in length. The genome of MATE 2, consisting of a G + C content of 52.1%, which is similar to the available genome sequences of *Xf* with G + C contents ranging from 51 to 52% (Castillo and Almeida, 2021) but significantly lower than those of sequenced *Xanthomonas* spp. (∼63.6 to 65.3%) (Ahern et al., 2014), suggests that the primary host of MATE 2 in the plant environment is *Xf*, as the G + C content of bacteriophages, in particular, is strongly correlated with that of their primary bacterial hosts (Cardinale and Duffy, 2011). Ninety-five open reading frames (ORFs) were identified in MATE 2 genome, of which only 33 (34.73%) are annotated with function with relatively high identity (>80% average amino acid identity), while the remaining 62 (65.26%) are with unknown functions. The functional genes highlighted on the genomic map (Figure 2) encode proteins involved in DNA replication, recombination, and modification; DNA packaging and structural proteins; and cell lysis. Based on the genome annotation, no known virulence, antibiotic resistance, or toxin-encoding genes were found within MATE 2 genome. Interestingly, genes encoding lysogeny-related proteins, such as integrase, are absent from the MATE 2 genome, indicating a strictly lytic infection cycle.

**Figure 3:**
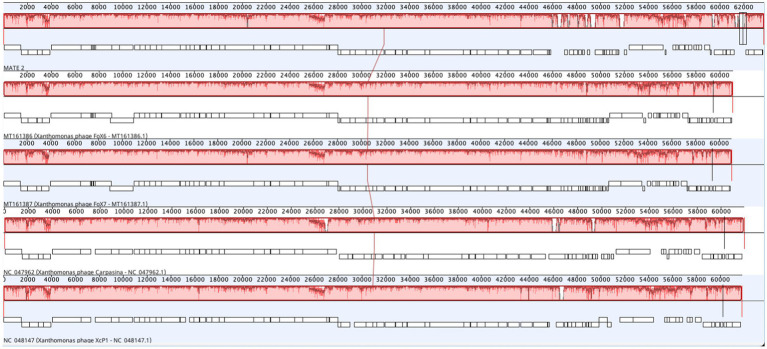
Schematic representation showing comparative genomes alignment of *Xylella* phage MATE 2 with *Xanthomonas* phage XcP1, *Xanthomonas* phage FoX6, *Xanthomonas* phage FoX7, and *Xanthomonas* phage Carpasina. Genome similarity is represented by a similarity plot within the colored blocks with the height of the plot proportional to the average nucleotide identity. The white regions represent sequence variations or fragments that were not aligned or contained sequence elements specific to a particular genome. The alignment was generated by Geneious Prime 2024.0.3.

**Figure 4:**
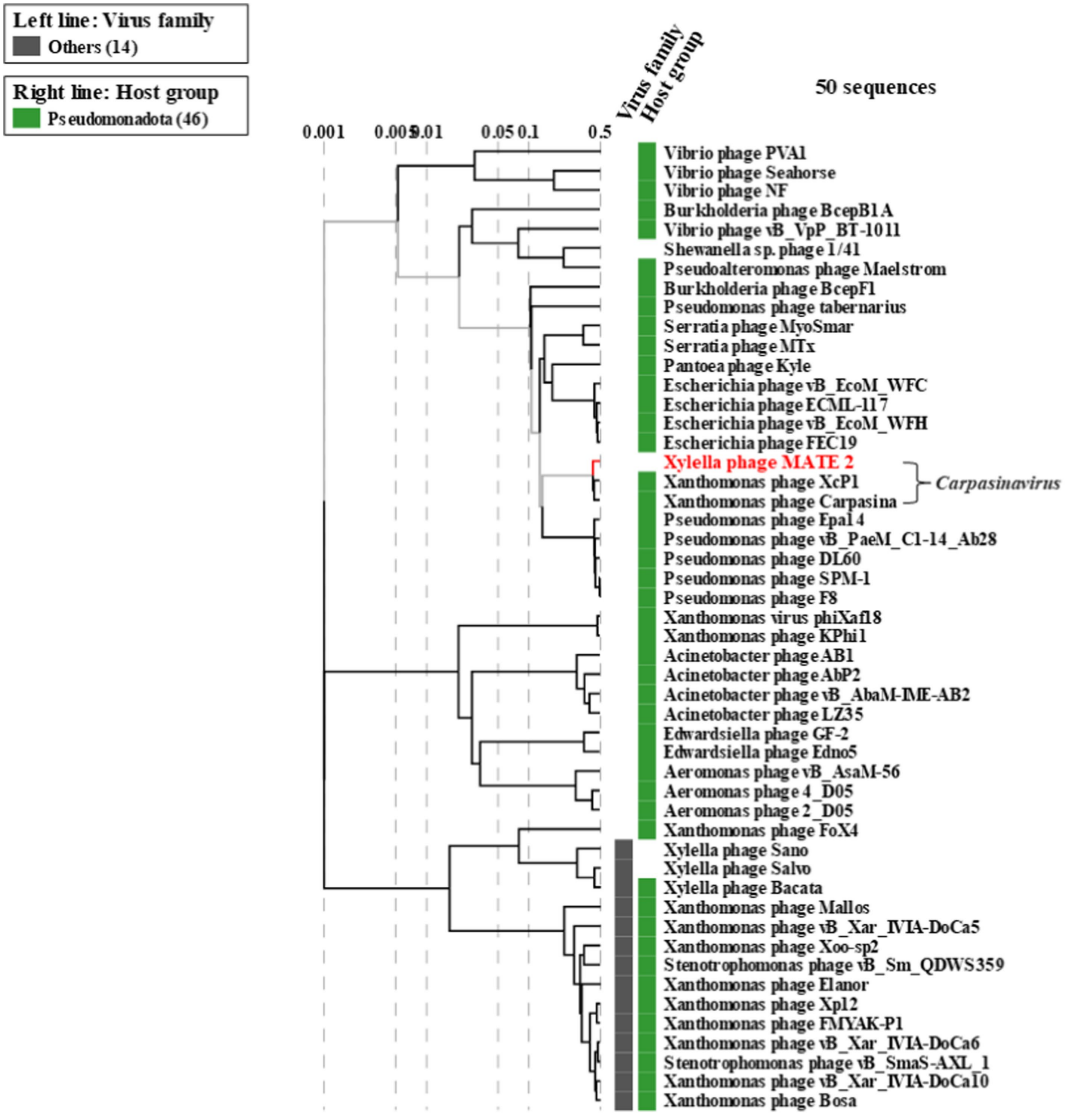
Proteomic tree of phage *Xylella* phage MATE 2 generated by ViPTree based on genome-wide sequence similarities computed by tBLASTx. *Xylella* phage MATE 2 belongs to the genus *Carpasinavirus*.

The genomic sequence analysis showed that MATE 2 shares maximum nucleotides identity with *Xanthomonas* phage XcP1 (88.2%; accession n°: NC_048147), followed by *Xanthomonas* phage FoX6 (86.9%; MT161386), *Xanthomonas* phage FoX7 (86.8%; MT161387), and *Xanthomonas* phage Carpasina (85.6%; NC_047962) (Figure 3). All these phages are members of genus *Carpasinavirus* and are known to infect *Xcc*. However, the identity found between MATE 2 and other related phages was lower than the demarcation criteria (95%) for the classification of new phage species, as per the “*International Committee on Taxonomy of Viruses*” (ICTV) rules. Hence, the name *Xylella* phage MATE 2 was proposed and the complete genome sequence was deposited in the GenBank under the accession number: PP816325. Furthermore, the proteomic tree of MATE 2 genome sequence and close homologs based on genome-wide sequence similarities computed by tBLASTx allocated MATE 2 in a clade with *Xanthomonas* phage XcP1 and *Xanthomonas* phage Carpasina classified in the genus *Carpasinavirus* (Figure 4); therefore, MATE 2 was considered as a tentative species of the genus *Carpasinavirus.*

### 3.3 Spot assay

**Figure 5:**
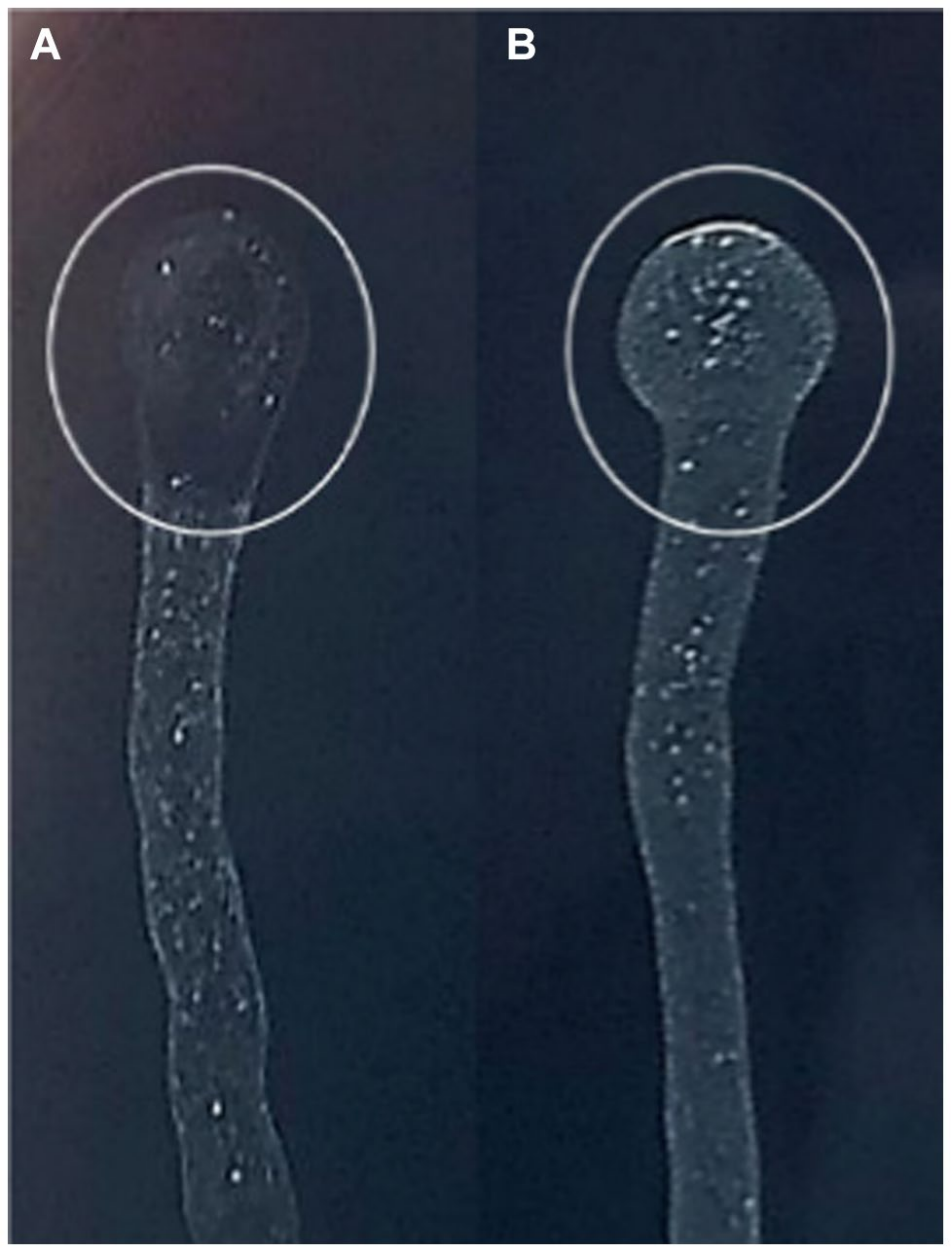
BYCE plate showing the lytic activity of *Xylella* phage MATE 2 against *Xylella fastidiosa* subsp. *pauca*. **(A)** *Xylella fastidiosa* treated with phage MATE 2. **(B)** *Xylella fastidiosa* treated with PBS. The circles indicate the treatment sites.

Phage sensitivity spot assay performed on *Xf* subsp. *pauca* culture showed that MATE 2 exhibits lytic activity against *Xf* cells. The lytic activity was highly accentuated, underscoring the effectiveness of MATE 2 phage in restraining *Xf* growth (Figure 5).

### 3.4 *Xylella fastidiosa* reduction assay

**Figure 6:**
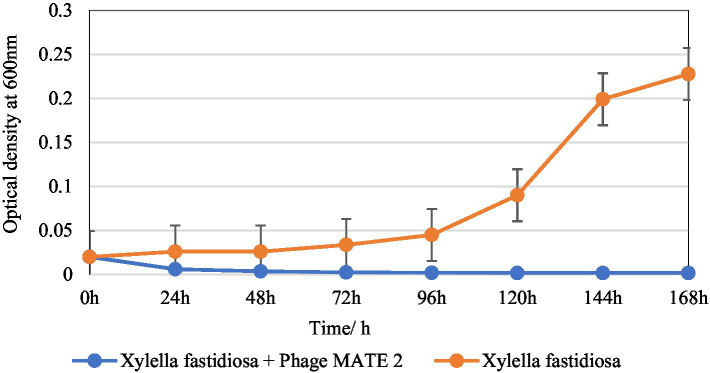
*In vitro* killing curve displaying the bacteriolytic effect of *Xylella* phage MATE 2 on *Xylella fastidiosa* subsp. *pauca* growth. The optical density of treated and untreated-bacterial culture with MATE 2 at 24-h interval for 7 days is compared. The bars indicate the error means for three replicates.

The inhibitory effect of MATE 2 on *Xf* growth was evaluated over a 7-day incubation period. The results showed a remarkable OD_600_ reduction in *Xf* culture after 24 h of treatment with MATE 2, resulting in 77% cell lysis (Figure 6). Upon treatment with MATE 2, the OD_600_ of the bacterial culture decreased significantly, leading to a total reduction of the *Xf* suspension after 48 h pi (Figure 6). The results also showed the exponential growth of untreated *Xf* bacteria after 72 h of incubation, while no growth was observed for the MATE 2-treated bacteria during the 7 days of incubation, suggesting no emergence of resistant bacteria within *Xf* population. This outcome shows that MATE 2 effectively inhibits the growth of *Xf*, indicating its potential implication for controlling *Xf*.

### 3.5 Electron and fluorescence microscopy evaluating MATE 2 activity against *Xylella fastidiosa*

**Figure 7:**
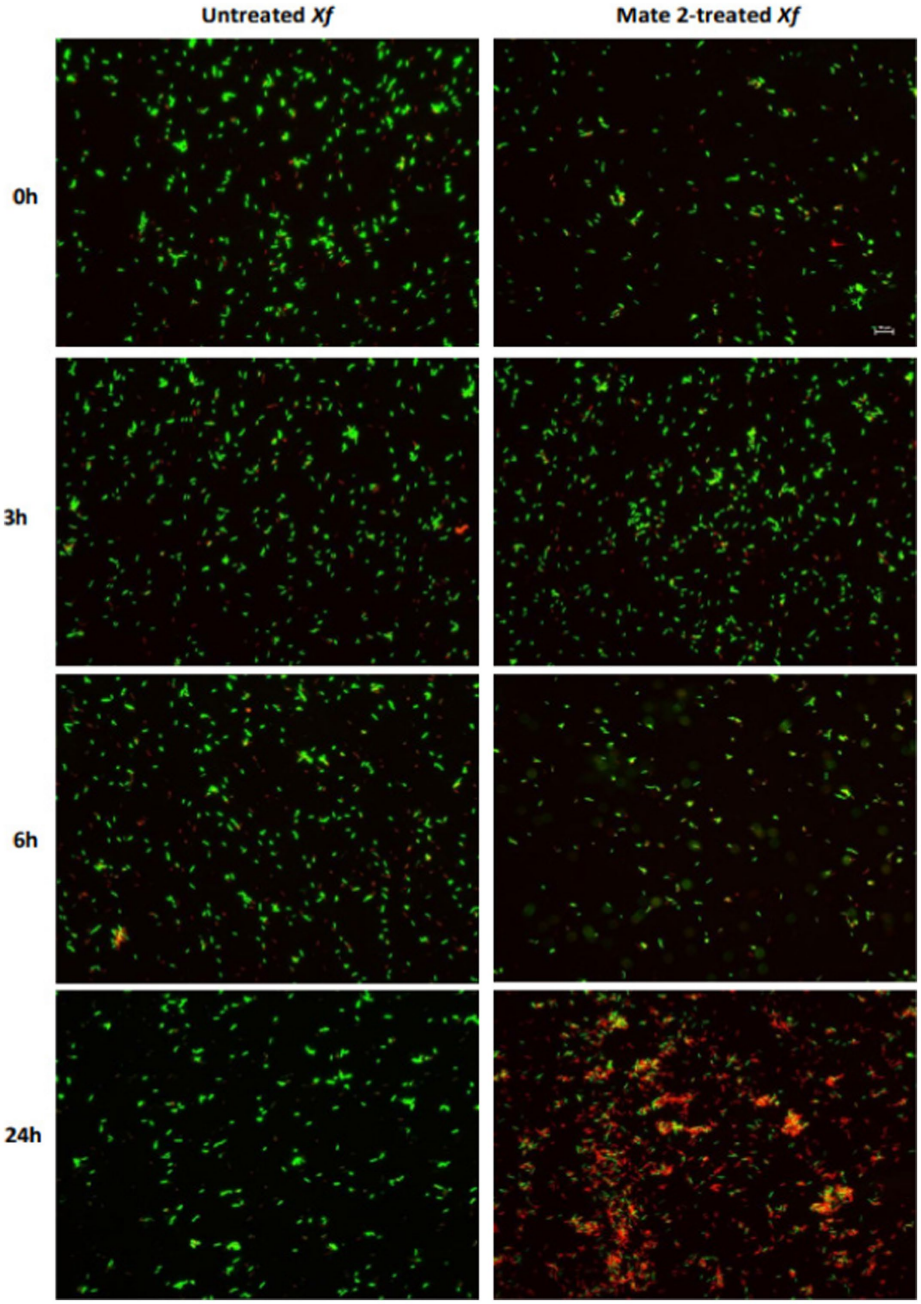
Fluorescent micrographs showing the effect of *Xylella* phage MATE 2 on *Xylella fastidiosa* cells during 24 h of incubation. Green and red fluorescence indicate live and dead cells, respectively. Magnification 2K×.

**Figure 8:**
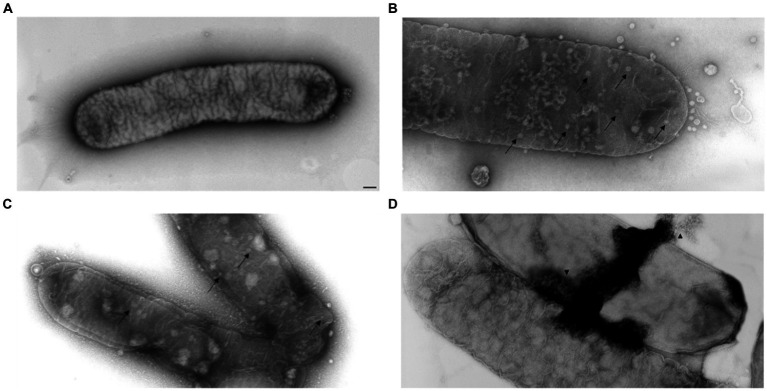
Transmission electron micrographs of *Xylella fastidiosa* cells challenged with *Xylella* phage MATE 2. **(A)** untreated *Xf* cells, used as control. **(B,C)** MATE 2-treated *Xf* cells ultrastructure with proliferation of phages (arrow). **(D)** Lysis of MATE 2-treated *Xf* cells with release of new phages (arrowhead). bar = 100 nm.

FM and TEM analyses were carried out to further examine the dynamics of phage MATE 2 virulence against *Xf*. FM micrographs showed that the staining of *Xf* cells shifted progressively from green (live bacterial cells) to red (dead bacterial cells) 3 h after the addition of MATE 2, indicating an increase in bacterial lysis (Figure 7). Additionally, an efficient cell death was observed after 24 h pi with MATE 2, confirming the results obtained from optical density measurements and validating its notable and rapid efficacy in lysing *Xf* cells. Analyzing the interaction between MATE 2 and *Xf* cells using TEM (Figure 8A), micrographs confirmed the attachment of MATE 2 particles to *Xf* cell surfaces 10 min after contact. Subsequently, after 30 min, phage progeny was already visible in *Xf* cytoplasm (Figures 8B,C) and the process ends with phage-induced *Xf*-cell lysis, which results in virion release (Figure 8D). This comprehensive examination provides profound insights into the *Xf-*MATE 2 interaction at an ultrastructural level, confirming the rapid lytic activity of phage MATE 2 against *Xf*.

### 3.6 Phage stability

**Figure 9:**
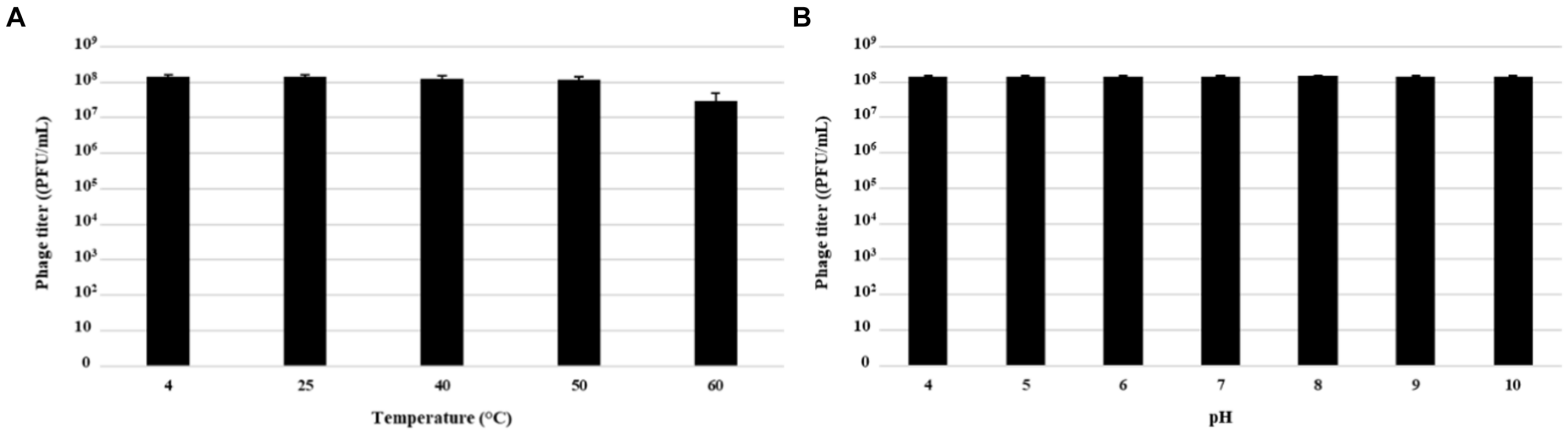
Histograms showing pH and thermal stability tests of *Xylella* phage MATE 2. **(A)** Phage infectivity after being treated with different temperatures for 60 min. **(B)** Phage infectivity after incubation at different pHs ranging from 4 to 10 for 60 min. Phage titers were determined using double agar overlay method. Error bars represent standard deviations from three replicates.

Physical and chemical factors which influence phage survival and persistence, were assessed through stability tests by exposing MATE 2 to different temperatures and pHs to assess its suitability as a biocontrol agent for plant protection. The thermal and pH stabilities of MATE 2 were estimated by measuring variations in survival rates based on the number of plaque-forming units (PFU). The results showed that MATE 2 is resistant to temperatures ranging from 4°C to 60°C, but at 60°C, the phage infectivity was reduced by about tenfold (Figure 9A). Furthermore, MATE 2 showed to have stable infectivity at pH ranging from 4 to 10 (Figure 9B).

## 4 Discussion

The considerable challenges in controlling *Xf* infections have prompted an urgent need for new antibacterial tools that can effectively combat this phytopathogen while preserving the environment and maintaining biodiversity. To this end, phage biocontrol provides a targeted and environmentally sustainable option to contrast *Xf* infections. The environmental benefits of phage therapy, including its low impact on non-target organisms and ecosystem integrity, highlight its potential as a biocontrol strategy or as part of integrated pest management strategies. However, the limited number of lytic phages identified and tested against *Xf* subsp. *pauca* poses a barrier to the adoption of phages for controlling this bacterium. With this aim, this study reports the isolation and characterization of a novel lytic bacteriophage, i.e., *Xylella* phage MATE 2, that showed therapeutic potential against *Xf* subsp. *pauca*.

*Xylella* phage MATE 2 was isolated from wastewater using a successful optimized isolation procedure for targeting and selecting rapid phages adsorbing to *Xf* cells. The genome analysis showed that MATE 2 is a new species in the genus *Carpasinavirus* within the class *Caudoviricetes*. Prediction of genes functions in the MATE 2 genome also showed the absence of genes encoding virulence factors, antibiotic resistance, toxin, or genes related to any lysogenic action, indicating its suitability for phage therapy in agriculture.

For biocontrol purposes, bacteriophages are required to maintain stability over a wide range of pH and temperatures and be resistant to changes in environmental and ambient conditions. The MATE 2 phage appears to meet these criteria, as demonstrated by its stability at different temperatures and pH ranges. Furthermore, our phage MATE 2, in addition to its powerful activity against *Xf*, was able to target various phytobacteria of the *Xanthomonadaceae* family, namely *Xanthomonas albilineans* and *Xanthomonas campestris*, without adverse effects on the other beneficial bacteria tested. This ability stands as an attractive trait for agriculture application, allowing MATE 2 to be polyvalent. Polyvalent phages are characterized by their specificity to recognize various host receptors [outer membrane protein or lipopolysaccharide (LPS)] using the same tail fiber protein and by their efficient and rapid adaptation to novel host constraints (Hamdi et al., 2017). This attribute, coupled with its stability, suggests that MATE 2 could effectively inhabit the phyllosphere and exert its broad-spectrum antibacterial activity against its bacterial hosts, thereby enhancing crop protection and agricultural production.

Lysis potential of MATE 2 against *Xf* subsp. *pauca* was investigated through a series of assays and microscopy analyses. Results of bacterial growth reduction assays demonstrated the effectiveness of MATE 2 in contrasting *Xf*, with considerable lytic activity. Indeed, MATE 2 was able to completely reduce and inhibit the growth of *Xf* for 7 days, highlighting its potential as a realistic predator of *Xf*. This tremendous lytic activity of MATE 2 against *Xf* was substantiated by TEM analyses, showing rapid adsorption of MATE 2 onto *Xf* cells and completion of its replication cycle in less than 1 h. This short duration between MATE 2 infection and *Xf* cell lysis, together with the slow growing propriety of *Xf*, makes MATE 2-based biocontrol a viable approach to combat *Xf* infections.

In the field of phage therapy research, the phage-encoded enzyme (i.e., endolysin) that can specifically hydrolyze the bacterial cell wall and quickly kill bacteria, is the most effective and widespread bactericidal agent on the planet (Liu et al., 2023). The high specificity of endolysins against the genus or species infected by the phage from which they originate is one of the main advantages of endolysins over conventional broad-spectrum antibacterial drugs (Mondal et al., 2020). In our instance, genomic analysis revealed that MATE 2 possesses an endolysin (Figure 2), which can also be used against *Xf.* Furthermore, recent findings showed that phages exhibit anti-biofilm properties against various pathogenic bacteria, including *Escherichia coli*, *Enterococcus faecium*, and *Enterococcus faecalis* (Melo et al., 2019; Chaudhary et al., 2022), as well as the capacity to act synergistically with other natural antimicrobials, such as bacteriocins and natural antimicrobial peptides (Barache et al., 2024). These findings encourages the exploration of MATE 2’s efficacy and its endolysin against *Xf* biofilm within the plant vascular system and their potential synergies with existing therapeutic agents to develop a more robust and integrated *Xf* disease control strategy.

In conclusion, the properties demonstrated by the novel isolated phage, namely lack of lysogenic content on its genome, stability at different temperatures and pH ranges, broad lytic spectra, and strong lytic activity against *Xf* suggest its realistic biocontrol use. Lastly, further studies are needed to explore the *in-planta* efficacy of MATE 2.

## Data availability statement

The datasets presented in this study can be found in online repositories. The names of the repository/repositories and accession number(s) can be found in the article/supplementary material.

## Author contributions

MS: Conceptualization, Data curation, Formal analysis, Investigation, Methodology, Software, Validation, Visualization, Writing – original draft, Writing – review & editing. KE: Conceptualization, Data curation, Investigation, Methodology, Software, Validation, Visualization, Writing – original draft, Writing – review & editing. OC: Data curation, Formal analysis, Investigation, Methodology, Software, Visualization, Writing – review & editing. AD: Data curation, Formal analysis, Investigation, Methodology, Software, Validation, Visualization, Writing – review & editing. FV: Data curation, Formal analysis, Methodology, Validation, Visualization, Writing – review & editing. TE: Conceptualization, Data curation, Formal analysis, Funding acquisition, Investigation, Methodology, Project administration, Resources, Software, Supervision, Validation, Visualization, Writing – original draft, Writing – review & editing.

## Footnotes

^1^ http://www.inphatc.com

^2^ http://www.genomicepidemiology.org/
